# A Set of Grand Challenges for Veterinary Regenerative Medicine

**DOI:** 10.3389/fvets.2016.00020

**Published:** 2016-03-04

**Authors:** Jennifer G. Barrett

**Affiliations:** ^1^Marion duPont Scott Equine Medical Center, Department of Large Animal Clinical Sciences, Virginia-Maryland College of Veterinary Medicine, Virginia Tech, Leesburg, VA, USA

**Keywords:** stem cells, regenerative medicine, veterinary medicine, tissue engineering, platelet-rich plasma

## Introduction

“Regenerative medicine” refers to therapies that aim to restore normal form and function using the body’s own biological machinery, such as stem cells and biologics. The use of regenerative medicine to treat injury and disease offers new hope to cure previously frustrating diseases and conditions. However, commercialization of biologic therapies in veterinary medicine has outpaced the speed of clinical research, and we are at risk of harming the reputation and standing of regenerative medicine before it has been fully optimized and developed. In response to this, international regulation of Veterinary Regenerative Medicine is underway. This highlights the need and opportunity for veterinarians and veterinary researchers to influence the optimal development of this field by working toward standardized approaches and evidence-based medicine.

The Frontiers journal approach to publication allows a more collaborative environment and enables a more sophisticated discussion about emerging challenges that need to be addressed in our work. My vision for the Veterinary Regenerative Medicine specialty section of *Frontiers in Veterinary Science* is to facilitate the timely publication of high-quality research articles in Veterinary Regenerative Medicine and to host discussion about provocative topics in our field. As the first journal with a section focused on Veterinary Regenerative Medicine, *Frontiers in Veterinary Science* has the opportunity of providing a much-needed forum for these timely topics.

Emerging challenges that are relevant to the development of safe and efficacious regenerative therapies for veterinary patients include the proper validation of reagents used in veterinary research, standardization of protocols and nomenclature, and highlighting the limitations as well as clinical relevance of research findings. Furthermore, another challenge to the field is merging complex processes and unique techniques associated with cell culture with a medical field that has established treatments and surgeries that may be used alongside regenerative therapies. This makes optimizing therapeutic approaches more complex, costly, and time consuming in the veterinary medical field where achieving evidence-based medicine is already challenging. Finally, advances in Veterinary Regenerative Medicine should be relevant and available to those studying human disease and basic science if it is to have its most profound impact. The open access format for Veterinary Regenerative Medicine offers a venue for dissemination of information about veterinary therapeutics that can accelerate translational medicine and the concept of “One Health.”

## Regulation of Veterinary Regenerative Medicine

In the European Union, the United States, Japan, and other countries, the regulatory framework for the therapeutic use of stem cells for human patients is well established (1). In contrast, the veterinary medical field has taken longer to develop these frameworks. In the United States, the Center for Veterinary Medicine (CVM) within the U.S. Department of Health and Human Services’ Food and Drug Administration (FDA) has initiated steps to regulate the field of Veterinary Regenerative Medicine in parallel with the regulatory environment of human regenerative medicine therapies.

In June 2015, the FDA published guidelines on the regulation of veterinary cell therapy (2). While a comprehensive survey of the international regulation of Veterinary Regenerative Medicine is outside the scope of this article, key features to glean from this FDA document include understanding whether cell therapy for veterinary patients is treated similarly or differently from human patients, what type of cell therapy constitutes a drug, and, if it is a drug, what level of manufacturing practice is required. Specifically for the US, this guidance document broadly constitutes what cell therapies require FDA approval, namely, allogeneic and xenogeneic cell therapies, and autologous therapies that are more-than-minimally manipulated, or that are used in a non-homologous manner, or that are used in food-producing animals, or that are combined with other drugs or devices for therapy. Any cell therapy that falls under these broad characteristics requires opening an Investigational New Animal Drug (INAD) file for any client-owned animal research that is to be undertaken and commercial products require New Animal Drug Application (NADA) approval. CVM used many of the guidelines developed for human cellular therapy by the FDA Center for Biologics Evaluation and Research (CBER). Thus, definitions of terms, such as “autologous,” “minimally manipulated,” “homologous usage,” and “good manufacturing processes” could be applied similarly for both humans and animals. It is notable that since the FDA evaluates each product and its data independently, the guidance document cannot be used *a priori* to know whether a therapy will require FDA approval for clinical use.

Internationally, regulatory bodies may take a different approach and not apply similar standards from human to veterinary cell therapies. *Frontiers in Veterinary Science* welcomes review articles about the regulation of Veterinary Regenerative Medicine since this is such a critical and timely topic to practitioners and researchers alike. For those of us with interests in academic research regardless of nationality, validation of protocols and procedures is a critical step toward regulatory approval and effective and safe application of regenerative therapeutics. To bring Veterinary Regenerative Medicine to its full clinical potential, funding for studies both validating the laboratory-based aspects of regenerative medicine and the clinical safety and efficacy of treatments is critical.

## Validation of Reagents Used in Veterinary Research

The ability to conduct sophisticated cell and molecular biological research in veterinary species has entered a heyday. Since the sequencing of the human genome, the reduced cost and increased availability of sequencing facilities have enabled sequencing of many other species’ genomes, including the horse, dog, cat, and cattle (3–6). This, together with the ease of quantitative PCR techniques, has given veterinary researchers access to study a host of gene expression patterns in response to experimental manipulation. The caveat is that as veterinary clinical researchers, we need a deep understanding of the molecular biology behind the assays and an incomplete understanding of the pitfalls of the assays and requirements for validation can limit the quality of our research. One example is that relative gene expression reliability and validity rely upon the use of a stably expressed gene or panel of genes in whatever system is being studied (7). Few publications in our field validate or address the stability of reference genes prior to calculating relative gene expression (8, 9). The proper design and validation of quantitative PCR assays is another area in need of further work. In particular, the use of SYBR green qPCR over TaqMan without adequate optimization of the SYBR green assay can result in unreliable data (10, 11). The MGB probe within the TaqMan assays reduces the chance of spurious results; however, this assay is not dominant in the veterinary literature to date.

Antibody-based assays are exceptionally important in cell and molecular biology research, and this is one area of weakness for veterinary research, since antibody reagents have not been readily available for our species of interest. That being said, the number of reagents that have been tested for cross-reactivity in veterinary species has increased, and biotechnology companies are beginning to see more value in creating equine or canine-specific antibodies due to increased demand. The proper validation of antibodies for an assay is a time consuming and expensive procedure but it is integral to the validity of our research (12). Ideally, an antibody that immunoprecipitates the protein can be validated by mass spectroscopy. Another approach to validation is to identify that only one protein is recognized by an antibody *via* Western blot analysis, and that expression levels (either on Western blot, immunofluorescence, or flow cytometry) correlate to any knock-down, knock-out, or down- or upregulation experiments. Without access to genomic knock-outs and with limited availability of predesigned commercially available knock-down reagents, such as siRNA for veterinary species, this process is more difficult and costly. Moreover, it is important to note that Western blots recognize denatured or non-denatured but non-native protein configurations. Secondary, tertiary, and quaternary native structure of a protein might be critical for epitope recognition, and native structure is often important for epitope recognition and effective antibody reagents for flow cytometry and immunofluorescence.

These difficulties do not obviate the need for validation; they highlight that a cooperative and collaborative approach to reagent validation would be beneficial to the entire field. Some published experiments using antibody-based assays have not been reproducible; the Veterinary Regenerative Medicine specialty section can provide a forum for optimization and validation of antibody reagents. We are including a “Protocol” publication type to aid in the dissemination of the detailed information needed for veterinary researchers to successfully move our field forward.

## Standardization of Protocols and Nomenclature

In the human and rodent model literature, much effort has been made to standardize the nomenclature of stem cells, including mesenchymal stromal cells, hematopoietic stem cells, and embryonic stem cells (13, 14). In contrast, much of the veterinary literature lacks detailed characterization of stem cell lines presumably partly due to limited resources available to veterinary researchers, and partly due to lack of reagents available for veterinary species. To avoid researchers repeating protocols or approaches that are not dependable, we need to pool our knowledge about inconsistently reliable reagents and results that may not be reproducible in a cooperative forum. We are fortunate for the opportunity to investigate species differences in stem cell function, gene expression, and cell surface expression to better elucidate properties that contribute to effective regenerative therapies. Beyond the scientific literature, commercially available veterinary therapies use terminology that is not consistent with the guidelines present in the human literature. For example, referring to stromal vascular fraction cells as “stem cells” is not consistent with position statements on the human side. Similarly, platelet-rich plasma is a term that has been applied to platelet and leukocyte-rich plasma, and platelet-rich but plasma-reduced blood products. There has been considerable effort in human therapy to standardize nomenclature of blood-based therapies. For veterinary medicine, this has begun, but further clarification and consensus is needed. There are serious concerns that inconsistent or inaccurate labeling of regenerative medicine products could impact the public’s perception of the efficacy of stem cell therapies, as well as biologics.

Furthermore, protocols for stem cell isolation, characterization, and differentiation vary from laboratory to laboratory, and the response of stem cells to those protocols can vary based on both the species and the tissue of origin of the stem cells (15, 16). We invite articles that take a broad look at protocols in the literature and relate relative efficacy of differentiation to whether the protocols were optimized to the species and tissue of origin. As *Frontiers in Veterinary Science* is an open access journal, review articles, editorial commentary, and potentially consortium statements published on this topic could act as a primary source of reliable information available to whomever is searching for information about stem cell therapy for veterinary patients.

## Clinical Relevance of Research Findings

Regenerative medicine research aims to use the natural machinery in the body to improve the clinical outcomes for various diseases and conditions; however, much of the research needed to develop these clinical approaches can be difficult to interpret for clinicians. Another goal of this specialty section is to provide commentary and review articles highlighting the clinical relevance of recent research publications. Moreover, the open review process we use at Frontiers allows readers to see the reviewers’ critiques, which often highlight where more research is needed before a given treatment is accepted as both safe and efficacious for clinical use. The education of clinicians about the differences between cell therapies or even tissue engineered materials is a critical part of what we hope to do in this specialty section. Potential topics we could focus on include studies or articles that discuss collection of tissues from patients and the use of cell therapies for injection of lesions. At *Frontiers in Veterinary Science*, we also invite articles and editorials that accompany primary research articles to relate innovative research findings to veterinary clinical practice now and in the future.

## One Health and a Challenge for Veterinary Researchers

Open access journals allow the dispersal of information rapidly and broadly. For *Frontiers in Veterinary Science*, this allows basic scientists and researchers studying human disease and basic science to access the veterinary literature, which is often unavailable at universities that do not have a college of veterinary medicine. Publication of high quality Veterinary Regenerative Medicine research and communicating the field broadly may fertilize collaboration and interaction across human and veterinary medicine and science. Also, having all of Veterinary Regenerative Medicine, whether it refers to small animals or large animals in the same specialty section further promotes the concept of “One Health.” The field of regenerative medicine and stem cell biology is growing exponentially, and this trend has now included Veterinary Regenerative Medicine. Taking a broader look at the regenerative medicine field and teasing out literature in non-veterinary publications that relates to veterinary medicine is another potential benefit of this specialty section.

## Conclusion

In summary, the Veterinary Regenerative Medicine specialty section of *Frontiers in Veterinary Science* invites high quality primary research articles in Veterinary Regenerative Medicine and stem cell biology for submission, as well as review articles, editorials, cases reports, protocols, and retrospectives. We encourage the Associate Editors and Review Editors to support the mission of the specialty section by using an open, interactive, constructive, and rapid review process to develop and advance articles prior to publication. The last challenge I put forth is the development of Research Topics, which generate focused collections of articles on a specific topic important to Veterinary Regenerative Medicine.

## Author Contributions

The author confirms being the sole contributor of this work and approved it for publication.

## Conflict of Interest Statement

The author declares being on the Scientific Advisory Board of ReCellerate, Inc.
